# Treatment Patterns, Clinical Events, and Costs of Care for Patients With Triple Negative Metastatic Breast Cancer: A Retrospective US Claims Database Study

**DOI:** 10.36469/001c.144499

**Published:** 2025-11-06

**Authors:** Manali Bhave, Tiffany Traina, Simon M. Collin, Jenny Tse, Nazneen Fatima Shaikh, Dajun Tian, Aimee M. Near

**Affiliations:** 1 Winship Cancer Institute, Emory University School of Medicine, Atlanta, Georgia, USA; 2 Memorial Sloan Kettering Cancer Center, New York, New York, USA; 3 Oncology Outcomes Research, AstraZeneca Pharmaceuticals Ltd., Cambridge, UK; 4 Evidence Strategy, Genesis Research, Boston, Massachusetts, USA; 5 Real World Solutions, IQVIA, Durham, North Carolina, USA; 6 Real World Solutions, IQVIA, Boston, Massachusetts, USA

**Keywords:** breast cancer, triple-negative breast cancer, healthcare costs, treatment patterns, real-world data, adverse events

## Abstract

**Background:**

Chemotherapy is the main treatment for patients with metastatic triple-negative breast cancer (mTNBC) who are ineligible for immunotherapy. TNBC is associated with poorer treatment outcomes than other breast cancer subtypes.

**Objective:**

To evaluate treatment patterns, quantify real-world healthcare costs and assess the burden of clinical events of interest (CEIs) among US patients with mTNBC who did not receive immunotherapy.

**Methods:**

This retrospective study used IQVIA PharMetrics® Plus healthcare claims data. Treatment-based proxies were used to identify patients first diagnosed with mTNBC from March 2017 to September 2023. Treatment regimens, frequency and incidence of CEIs, and all-cause, breast cancer–related, and CEI-related costs per patient per month (PPPM, including drug costs) were described during overall follow-up (any line of therapy [LOT]) and during LOT1 and LOT2.

**Results:**

A total of 2717 patients with mTNBC (99.1% female; mean±SD age, 55.6 ± 10.7 years) were identified. Over the follow-up period (median [Q1,Q3], 11.7 [6.0, 26.5] months), most patients (73.1%) only reached LOT1, and the remaining 26.9% of patients had multiple LOTs. Most patients had chemotherapy in LOT1 (98.1%) and LOT2 (90.6%); 98.5% had chemotherapy across any LOTs. Taxanes were the most common, observed in 74.8% of the overall cohort, followed by anthracyclines (56.4%). Across any LOT, 76.1% of patients had ≥1 CEI, most commonly hematological (49.5%), gastrointestinal (44.3%), infusion-related reactions (31.2%), and fatigue (27.8%). Mean (SD) all-cause total costs PPPM were 14 245(12 776) overall (of which 79.6% were BC-related and 34.7% were CEI-related), 17 809(18 806) during LOT1, and 19 797(24 763) during LOT2.

**Discussion:**

Our study findings confirm previously reported high economic burden of mTNBC, with about 80% related to BC treatment. Most patients experienced CEIs during treatment, and these accounted for one-third of their total healthcare costs.

**Conclusions:**

Our study focused on patients with mTNBC with the greatest unmet need, namely those ineligible for immunotherapy. In patients with mTNBC, most of whom received chemotherapy as standard of care, CEIs presented both a clinical and economic burden, highlighting the need for newer treatments that balance total costs of care with adverse events and clinical benefit.

## BACKGROUND

Breast cancer (BC) is highly heterogeneous and is classified into several subtypes. Patients with a first or recurrent diagnosis of BC undergo a series of tests for human epidermal growth factor receptor 2 (HER2) and hormone receptor (HR) status to identify the BC subtype and determine the best course of treatment.^1,2^ Based on assessment of immunohistochemistry levels and in situ hybridization results, HER2 expression is recognized clinically as negative (encompassing “low,” “ultralow,” and “null” levels of expression) or positive. HR positivity is determined by presence of estrogen receptors and/or progesterone receptors. Triple-negative BC (TNBC) is negative for estrogen receptors, progesterone receptors, and HER2. TNBC is the most aggressive BC subtype, accounting for nearly 20% of all BC, with high recurrence rates and the lowest 5-year survival rate (78%) of the BC subtypes. Among patients with TNBC, 5-year survival reduces drastically with metastasis, from 92% with localized disease, to 67% with regional disease, and to 14% with distant metastasis.^3–5^ Patients with TNBC are more likely to have advanced cancer stages at diagnosis with a poorer prognosis than other BC subtypes.^6,7^ A recent systematic review reported the annual healthcare costs for metastatic TNBC (mTNBC) in the range of $100 000 to $300 000 per patient in the United States (US), indicating a significant economic burden.^8^

First-line treatment for mTNBC is currently chemotherapy, specifically, anthracycline or taxane-based regimens.^9^ Patients with programmed death ligand 1 (PD-L1) protein expression above a threshold, typically a combined positive score of at least 10, are eligible for first-line treatment with PD-1 and PD-L1 inhibitors (PD-L1 immunotherapy).^9,10^ However, patients who are PD-L1–negative, representing 60% to 80% of patients with mTNBC, and patients with contraindications such as autoimmune disease, are ineligible for immunotherapy.^11^ Beyond disease progression, treatment tolerability, especially for chemotherapy, is a key consideration for treatment decision-making because it is typically the major reason for treatment discontinuation or switching in patients with metastatic BC (mBC).^12–15^ This is a particularly critical problem in the treatment of mTNBC due to the limited therapeutic options. Furthermore, managing adverse events results in a high economic burden, adding to the already significant costs of mTNBC treatment.^8,16,17^ These factors, combined with overall poor outcomes for patients with mTNBC, indicate a need for more effective and tolerable therapies in the management of mTNBC.

As the therapeutic landscape for mTNBC continues to evolve, there is an increasing need for studies describing the costs of care and tolerability of treatment using recent data. To that end, this retrospective study evaluated real-world treatment patterns, costs of care and clinical events of interest (CEIs), including potential adverse events of treatment, among patients with mTNBC, focusing on those who are not candidates for PD-L1 immunotherapy, representing patients with the greatest unmet need for newer therapies.

## METHODS

This retrospective study used the IQVIA PharMetrics^®^ Plus patient-level claims database from 1 September 2016 to 31 December 2023. PharMetrics Plus® is a US health plan claims database comprised of fully adjudicated medical and pharmacy claims for more than 174 million unique enrollees since 2006, across the 4 US census regions. PharMetrics Plus® contains closed-source payer claims data; data contributors to the database are largely commercial health plans. It contains a longitudinal view of inpatient and outpatient services, prescription and office/outpatient administered drugs, costs, and detailed enrollment information. All data are compliant with the Health Insurance Portability and Accountability Act to protect patient privacy.

### Study Population

Patients with at least 1 *International Classification of Diseases, Tenth Revision, Clinical Modification* (ICD-10-CM) diagnosis code for BC and metastasis were identified in PharMetrics Plus® claims data from 1 March 2017 to 30 September 2023 (selection window). Selection criteria included continuous enrollment 6 months before (baseline) and at least 3 months after (follow-up) the index date (earliest metastasis code). Follow-up ended at the earliest of end of the fourth line of therapy (LOT 4), end of continuous enrollment, or end of the study period. In addition, patients were required to have evidence of at least 1 line of BC therapy during follow-up and no diagnosis codes for other cancers or metastasis within 15 months pre-index. A treatment-based proxy was used to establish TNBC status, whereby patients with claims for treatments indicated for HR+/HER2- breast cancer (CDK4/6 inhibitor, mTOR inhibitor, PIK3CA inhibitor, AKT inhibitor), HR+ breast cancer (endocrine therapy) or HER2+ breast cancer (trastuzumab, pertuzumab, margetuximab, ado-trastuzumab emtansine, lapatinib, neratinib, tucatinib) during the study period were excluded. Similar proxies have been used in previously published claims database studies, due to the lack of biomarker data or specific diagnosis codes for BC subtypes in claims.^18,19^ To identify by proxy those who were not candidates for PD-L1 immunotherapy, patients who received PD-L1 immunotherapy (atezolizumab/pembrolizumab) at any time during the study period were excluded.

### Study Measures

Treatment patterns were reported during the follow-up period where a LOT was defined as a continuous segment of medication use during follow-up. The start of the treatment was based on the prescription fill date or drug administration date and the initiation of a LOT was defined as the first occurrence of any anticancer drug on or after index date. A universal LOT regimen was made up of the first identified drug, and all other drugs received within the following 28 days. Any mBC treatment occurring between LOT 1 initiation date and 28 days from LOT initiation date was considered part of a combination therapy. The LOT advanced when a new drug was given that was not part of LOT 1, or when there was a treatment gap of more than 360 days or 180 days for the same drug if the regimen contained an oral drug or parenteral drug, respectively. Detailed rules for defining combination therapies and LOT advancements are detailed in **Supplemental Table S1**. Systemic treatment during LOT 1 and LOT 2 by treatment categories, and specific treatment regimens observed in LOT 1 and LOT 2, were reported.

A clinical event of interest (CEI) was defined as an unfavorable and unintended condition or side effect with onset during medical treatment that may or may not be considered related to the medical treatment.^20^ CEIs were based on the adverse events listed in the product labels for BC treatments. Given that the start and resolution dates for CEIs were not available in claims data, it was assumed that the earliest instance of the diagnosis code for the CEI during a LOT corresponded to the first instance of the CEI. Any diagnosis code for the same category of CEI within a 14-day window was considered as part of the same CEI episode. After the 14-day window, the patient was considered at risk for another occurrence of the CEI.^21^ The CEIs assessed, based on diagnosis codes, included hematological events (eg, anemia, leukopenia, neutropenia), gastrointestinal events (eg, constipation, diarrhea, nausea, stomatitis/mucositis), hepatic events (eg, elevation of liver enzymes or bilirubin), alopecia, interstitial lung disease (ILD), pneumonitis, ocular events (eg, conjunctivitis, dry eyes, vision loss, photopsia), fatigue, infusion-related reaction (IRRs),^22^ rash, hyperglycemia, and sinus bradycardia. For chronic conditions (alopecia, ILD, sinus bradycardia), patients could not have multiple occurrences of the CEI. Therefore, the number of discrete occurrences per patient was a maximum of 1 during follow-up. Patients with these chronic conditions during baseline were not considered as at risk for the same condition during follow-up and patients with diabetes during baseline were not considered at risk for hyperglycemia during follow-up. CEIs were reported as frequencies (number of discrete occurrences of CEI per patient) and incidence of discrete occurrences of CEIs (rate per 1000 person-years) during any LOT, LOT 1, and LOT 2. Time to first CEI event (days) was defined as the time from the start of any LOT to the first evidence of CEI during overall treated period. Time to first CEI was also calculated separately from start of LOT1 and from start of LOT2.

All-cause, BC-related, and CEI-related healthcare costs were reported over the variable follow-up period. BC-related costs were identified from claims with BC diagnosis codes in any position or BC treatments, including treatments for CEIs. CEI-related costs (a subset of BC-related costs) were identified using claims containing a diagnosis code for BC in any position with either claims containing a diagnosis code for a CEI in any position, or claims related to CEI treatment or prophylaxis. All-cause healthcare costs in the variable follow-up period were also reported stratified by the presence or absence of CEI within 12 months of LOT initiation and by LOT. CEI-related healthcare costs per 14-day CEI event during the treated follow-up (ie, during LOTs) were also reported. Costs were standardized as per patient per month (PPPM), calculated as total costs during the follow-up period divided by the number of months in the follow-up period to account for the variable time periods. Costs were converted to 2024 US dollars using the medical component of the Consumer Price Index.

### Statistical Analysis

Descriptive statistics were produced for all relevant study measures previously described. Categorical measures were presented using frequency (number and percentage) of total study patients observed in each category. Continuous and count variables were described using mean, SD, median, first quartile (Q1), and third quartile (Q3). Time to CEI was calculated only for patients who experienced a CEI during any LOT or, separately, during LOT1 or LOT2. All analyses were based on observed, not projected, data. Analyses were conducted using SAS^®^ Release 9.4 (SAS Institute Inc.).

## RESULTS

Of 113 778 patients with evidence of mBC during the selection window, a total of 2717 patients met the selection criteria and were included in the mTNBC cohort (**Supplemental Figure S1**). The mean (SD) age of the study cohort was 55.6 (10.7) years, with more than two-thirds of patients at least 50 years of age at index. Nearly all patients (>99%) were female, with the highest proportion of patients located in the US South (42.5%) followed by the Midwest (27.4%). Most patients (90.8%) had commercial or self-insured payer types and preferred provider organization plans were the most common (67.8%). The distribution of index years was similar from 2017-2023, ranging from 9.8% to 16.7% indexed in each year (**Table 1**).

**Table 1.** Baseline Demographic and Clinical Characteristics of the mTNBC Cohort

**Measures**	**mTNBC Cohort (N = 2717)**
Age (years), continuous	
Mean (SD)	55.6 (10.7)
Median (Q1, Q3)	56 (48, 63)
Age categories (years), n (%)	
18-34	67 (2.5)
35-44	335 (12.3)
45-54	801 (29.5)
55-64	1068 (39.3)
65-74	322 (11.9)
75+	124 (4.6)
Female, n (%)	2693 (99.1)
Geographic region, n (%)	
South	1155 (42.5)
Midwest	745 (27.4)
Northeast	444 (16.3)
West	365 (13.4)
Unknown	8 (0.3)
Payer type, n (%)	
Commercial or self-insured	2467 (90.8)
Medicare risk	219 (8.1)
Medicaid	14 (0.5)
Other/unknown^a^	17 (0.6)
Plan type, n (%)	
Preferred provider organization	1841 (67.8)
Health maintenance organization	540 (19.9)
Point of service	174 (6.4)
Consumer directed health care	115 (4.2)
Indemnity	28 (1.0)
Other/unknown^b^	19 (0.7)
Index year, n (%)	
2017 (beginning March 1)	454 (16.7)
2018	453 (16.7)
2019	413 (15.2)
2020	346 (12.7)
2021	332 (12.2)
2022	267 (9.8)
2023 (through September 30)	452 (16.6)
National Cancer Institute comorbidity index, continuous	
Mean (SD)	0.1 (0.2)
Median (Q1, Q3)	0 (0, 0)
National Cancer Institute comorbidity index, categorical, n (%)	
0	2281 (84.0)
>0 to <1	391 (14.4)
1 to <2	45 (1.7)
2 to <3	0 (0.0)
3+	0 (0.0)
Specific comorbidities, n (%)^c^	
Obesity (based on diagnosis codes)	501 (18.4)
Diabetes	228 (8.4)
Chronic pulmonary disease	106 (3.9)
Rheumatologic disease	58 (2.1)
Congestive heart failure	46 (1.7)
Liver disease	33 (1.2)
Renal disease	32 (1.2)
Peripheral vascular disease	15 (0.6)
Sites of metastasis, using diagnosis codes on the index date (not mutually exclusive), n (%)	
Regional (lymph nodes, breast)	2212 (81.4)
Other (skin, bone and bone marrow, unspecified sites)	358 (13.2)
Distant visceral (liver, lung, other visceral sites)	269 (9.9)
Distant CNS (brain and other CNS)	87 (3.2)
Treatment during baseline, n (%)	
Chemotherapy	354 (13.0)
Targeted therapy	17 (0.6)
Radiation therapy	31 (1.1)

Abbreviations: CNS, central nervous system; mTNBC, metastatic triple-negative breast cancer; Q1, first quartile; Q3, third quartile.

^a^In payer type, other/unknown may include Medicare Part C, SCHIP, Medicare Cost (Medicare Supplemental), pharmacy benefit only, unknown/missing, and no enrollment.

^b^In plan type, other/unknown may include health savings account, unknown/missing, and no enrollment.

^c^Comorbidities were identified using diagnosis codes. Conditions present in <1% of the cohort are not shown.

Most patients (84.0%) had a baseline National Cancer Institute (NCI) comorbidity index score of 0. The most common comorbidities were obesity (18.4%), diabetes (8.4%), and chronic pulmonary disease (3.9%). Most patients had evidence of regional metastasis (81.4%), while distant visceral (9.9%) and distant CNS (3.2%) metastases were less common. During the baseline period, 13.0% of the patients had chemotherapy. Outcomes were assessed over the variable follow-up period with a mean (SD) and median (Q1, Q3) follow-up of 19.6 (18.6) and 11.7 (6.0, 26.5) months, respectively.

Most patients (73.1%) only reached the first LOT during follow-up, and the remaining 26.9% of patients had multiple LOTs, with only 7.3% of patients having 3 or more LOTs. The mean (SD) duration of LOT 1 was 113.0 (113.3) days and LOT 2 was 119.6 (119.1) days. Almost all patients (98.5%) had chemotherapy and 7.5% of patients had targeted therapy, including PARP inhibitors, VEGF inhibitors, and sacituzumab govitecan, during follow-up. Between individual LOTs, there was variation in the observed chemotherapy classes. For example, taxanes were observed in 69.8% of LOT 1 and 26.4% of LOT 2 regimens. While 12.5% of LOT 1 regimens contained cytotoxic therapy, this increased to 47.3% in LOT 2. Targeted therapies were also more frequently observed in LOT 2 than in LOT 1, particularly poly (ADP-ribose) polymerase (PARP) inhibitors (6.0% vs 1.3%) and sacituzumab (4.2% vs 0.3%). The 5 most common monotherapy and combination therapy treatment regimens during LOTs 1 and 2 are described in **Table 2**.

**Table 2.** Treatment Patterns and Top 5 Regimens for the mTNBC Cohort and by Line of Therapy

**Measures**	**Overall mTNBC Cohort (N = 2717)**	**During LOT 1 (N = 2717)**	**During LOT 2 (N = 731)**
Chemotherapy, n (%)	2677 (98.5)	2666 (98.1)	662 (90.6)
Taxane	2031 (74.8)	1897 (69.8)	193 (26.4)
Other chemotherapy^a^	1907 (70.2)	1766 (65.0)	223 (30.5)
Anthracyclines	1532 (56.4)	1376 (50.6)	218 (29.8)
Cytotoxic chemotherapy	685 (25.2)	339 (12.5)	346 (47.3)
Platinum-based chemotherapy	546 (20.1)	450 (16.6)	94 (12.9)
Targeted therapy, n (%)^b^	204 (7.5)	70 (2.6)	90 (12.3)
PARP inhibitors	97 (3.6)	34 (1.3)	44 (6.0)
Sacituzumab govitecan-hziy^c^	72 (2.6)	8 (0.3)	31 (4.2)
VEGF inhibitors	41 (1.5)	25 (0.9)	12 (1.6)
LOT 1 treatment regimens (top 5)			
Cyclophosphamide + doxorubicin + paclitaxel combination therapy		930 (34.2)	
Cyclophosphamide + docetaxel combination therapy		304 (11.2)	
Cyclophosphamide + doxorubicin combination therapy		265 (9.7)	
Paclitaxel monotherapy		247 (9.1)	
Capecitabine monotherapy		222 (8.2)	
LOT 2 treatment regimens (top 5)			
Capecitabine monotherapy			202 (27.6)
Cyclophosphamide + doxorubicin combination therapy			98 (13.4)
Cyclophosphamide + doxorubicin + paclitaxel combination therapy			80 (10.9)
Cisplatin + gemcitabine combination therapy			39 (5.3)
Paclitaxel monotherapy			38 (5.2)

Abbreviation: LOT, line of therapy; mTNBC, metastatic triple-negative breast cancer; PARP, poly (ADP-ribose) polymerase inhibitor; VEGF, vascular endothelial growth factor.

^a^Other chemotherapy includes cyclophosphamide, ixabepilone, fluorouracil, and methotrexate.

^b^There were no patients with NTRK or RET inhibitors during follow-up.

^c^Sacituzumab govitecan was considered as a treatment for mTNBC if the claim occurred before February 3, 2023, when it was approved for HR+ HER2- metastatic breast cancer (accelerated approval for mTNBC was granted on April 22, 2020, followed by regular approval on April 7, 2021).

**Figure 1.**
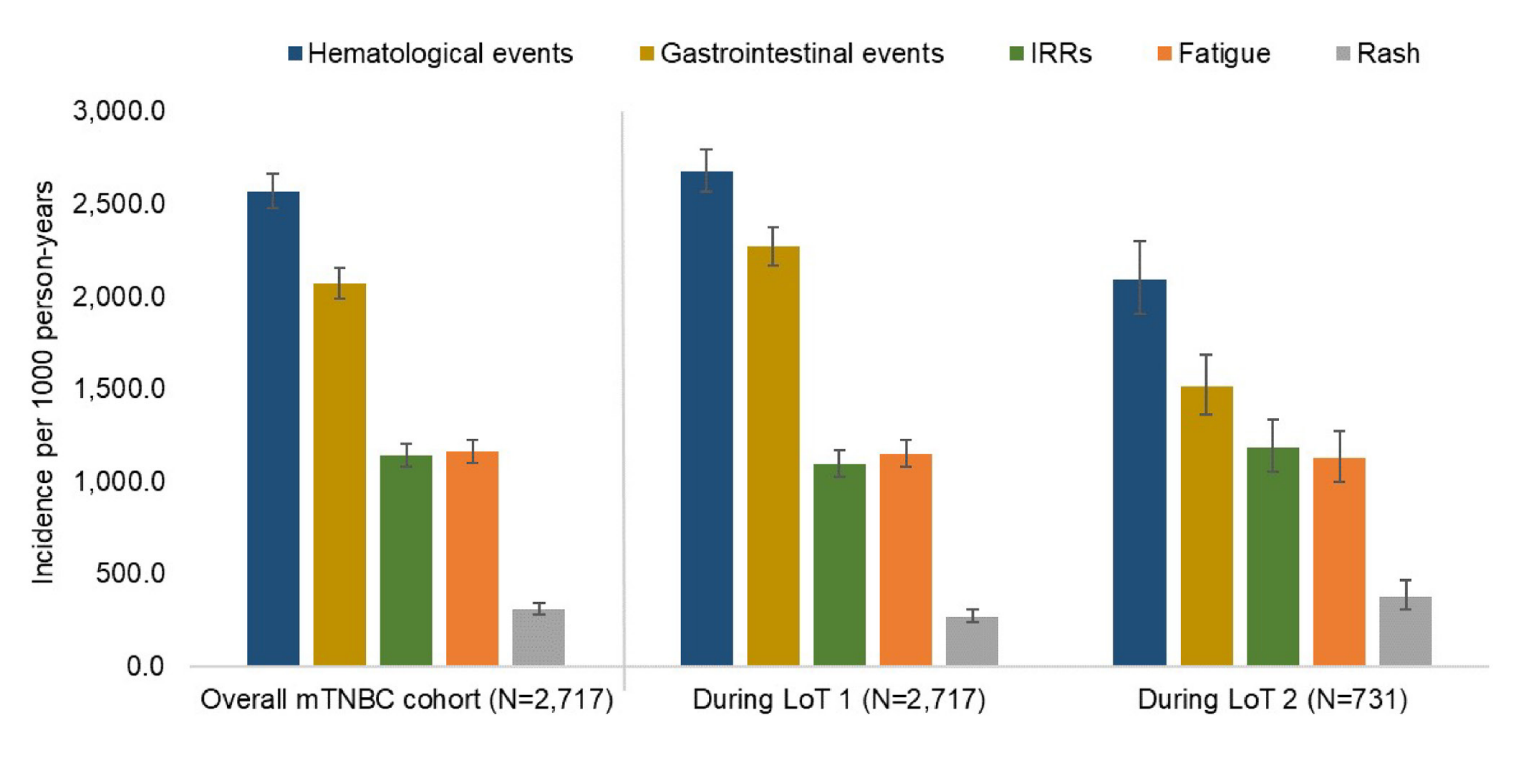
Incidence Rates of the Most Frequently Observed CEIs (Top 5) for the mTNBC Cohort and by LOT During Treated Follow-up

Abbreviations: CEI, clinical events of interest; mTNBC, metastatic triple-negative breast cancer; LOT, line of therapy; IRRs, infusion-related reaction.

The top 5 CEIs by incidence rate during any LOT, LOT 1, and LOT 2 were hematological events, gastrointestinal events, IRRs, fatigue, and rash (**Table 3, Figure 1**). ILD, pneumonitis, and sinus bradycardia were observed in less than 1% of patients during any LOT. Among patients with CEIs, the median (Q1, Q3) time from any LOT start to CEI was shortest for hematological (14 [1, 46] days) and gastrointestinal events (14 [0, 41] days) (**Supplemental Table S2**). For the most common CEIs, there was some variation in the median time to event during LOT 1 vs LOT 2 (**Supplemental Figure S2**); however, these differences did not exceed 2 weeks. The CEI with the longest median (Q1, Q3) time to event was ILD (83 [27, 123] days) during any LOT, followed by ocular events (61 [25, 105] days). At least 1 CEI was observed in 83.7% (2273/2717) of patients within 12 months of any LOT initiation; from LOT 1 or LOT 2 initiation, 82.8% (2251/2717) and 81.9% (599/731) of patients, respectively, experienced at least 1 CEI within 12 months.

**Table 3.** Proportion of Patients and Incidence Rates of CEIs for the mTNBC Cohort and by LOT During Treated Follow-up

**CEI**	**Overall mTNBC Cohort (N = 2717)**			**During LOT 1 (N = 2717)**			**During LOT 2 (N = 731)**		
	**No. at Risk^a^**	**Patients With ≥1 CEI, n (%)**	**Incidence Rate per 1000 PY (95% CI)**	**No. at Risk**	**Patients With ≥1 CEI, n (%)**	**Incidence Rate per 1000 PY (95% CI)**	**No. at Risk**	**Patients With ≥1 CEI, n (%)**	**Incidence Rate per 1000 PY (95% CI)**
Alopecia^b^	2671	68 (2.5)	58.0 (45.7–73.5)	2671	57 (2.1)	68 (52.5–88.2)	709	7 (1.0)	29.9 (14.3–62.8)
Fatigue	2633	732 (27.8)	1163.6 (1102.9–1227.8)	2618	579 (22.1)	1149.6 (1078.5–1225.5)	682	169 (24.8)	1127.7 (997.5–1275.0)
Gastrointestinal events	2616	1158 (44.3)	2068.6 (1986.5–2153.9)	2584	1018 (39.4)	2269.0 (2167.9–2374.9)	662	191 (28.9)	1515.0 (1361.3–1685.9)
Stomatitis /mucositis	2713	158 (5.8)	166.8 (145.2–191.6)	2713	136 (5.0)	196.0 (168.4–228.1)	724	20 (2.8)	104.1 (70.3–154.1)
Hematological events	2543	1260 (49.5)	2569.6 (2476.2–2666.5)	2497	1097 (43.9)	2676.7 (2565.0–2793.3)	621	227 (36.6)	2092.5 (1904.8–2298.7)
Hepatic events	2714	59 (2.2)	69.1 (55.8–85.7)	2712	42 (1.5)	62.2 (47.5–81.5)	728	11 (1.5)	62.0 (37.4–102.8)
Hyperglycemia^c^	2281	109 (4.8)	155.7 (133.2–181.9)	2276	82 (3.6)	155.0 (128.7–186.7)	600	26 (4.3)	147.1 (102.8–210.3)
ILD^b^	2706	11 (0.4)	9.2 (5.1–16.6)	2706	7 (0.3)	8.2 (3.9–17.3)	723	2 (0.3)	8.3 (2.1–33.1)
IRR	2585	806 (31.2)	1143.2 (1082.1–1207.7)	2529	632 (25.0)	1096.8 (1026.3–1172.2)	676	181 (26.8)	1186.9 (1053.4–1337.2)
Ocular events	2710	170 (6.3)	179.4 (157.0–205.1)	2708	127 (4.7)	184.9 (158.1–216.2)	729	32 (4.4)	148.4 (107.0–205.7)
Pneumonitis	2717	0 (0.0)	No events	2717	0 (0.0)	No events	731	0 (0.0)	No events
Rash	2708	233 (8.6)	311.6 (281.6–344.9)	2704	174 (6.4)	271.0 (238.1–308.4)	724	62 (8.6)	380.6 (310.3–466.9)
Sinus bradycardia^b^	2651	18 (0.7)	15.4 (9.7–24.4)	2651	13 (0.5)	15.6 (9.1–26.9)	712	4 (0.6)	16.9 (6.4–45.1)

Abbreviations: CEI, clinical events of interest; CI, confidence interval; LOT, line of therapy; PY, person-year; ILD, interstitial lung disease; IRR, infusion-related reaction; mTNBC, metastatic triple-negative breast cancer.

^a^Number of patients at risk for specific CEIs differed from the overall cohort size because, if the patient had evidence of the chronic condition during baseline or if the patient had evidence of the CEI within a 14-day window of LOT 1 initiation, they were considered not at risk.

^b^For chronic conditions (alopecia, ILD, sinus bradycardia), patients could not have multiple occurrences of the CEI. Patients with these conditions during baseline were not considered as at risk during follow-up.

^c^Patients with diabetes during baseline were not considered as at risk for hyperglycemia.

**Figure 2.**
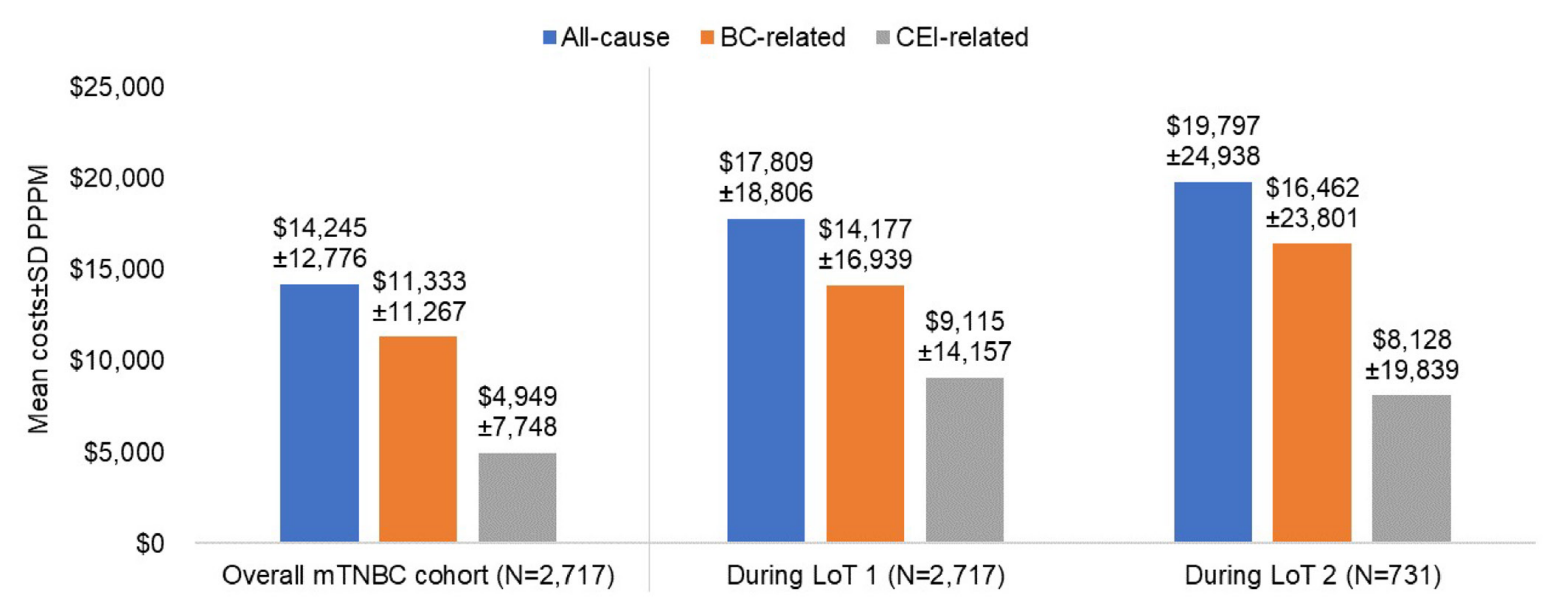
All-Cause, BC-Related, and CEI-Related Total Costs per Patient per Month in the mTNBC Cohort During Follow-up (Minimum 3 Months) and During First and Second Lines of Therapy Abbreviations: CEI, clinical events of interest; mTNBC, metastatic triple-negative breast cancer; LOT, line of therapy; IRRs, infusion-related reaction.

During follow-up, mean (SD) all-cause healthcare costs PPPM were $14 245 ($12 776) in the study cohort (**Figure 2**). BC-related and CEI-related costs accounted for 79.6% and 34.7% of mean total costs, respectively. Outpatient medical costs were the largest contributors to all-cause (73.4%), BC-related (77.3%), and CEI-related (54.4%) mean costs PPPM. Further breakdown among the subgroups of outpatient medical costs is described in **Supplemental Table S3**. Although inpatient costs contributed to 18% of mean all-cause and BC-related costs, they accounted for 37% of mean CEI-related costs. CEI-related costs accounted for a larger proportion of all-cause costs in LOT 1 (51.2%) and LOT 2 (41.1%), than during the entire follow-up (34.7%).

**Figure 3.**
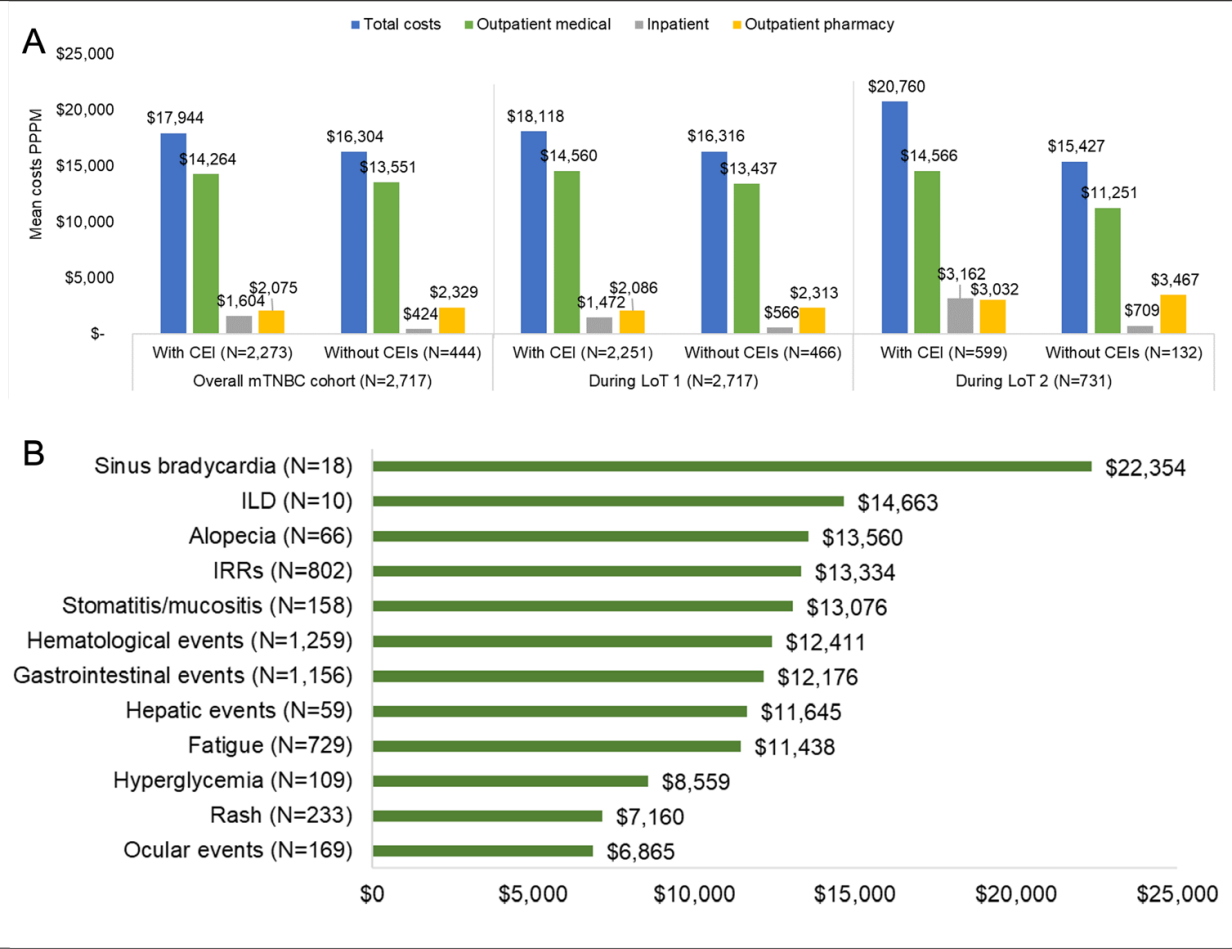
All-Cause Costs by Evidence of CEIs Within 12 Months of LOT Initiation (**A**) and Mean CEI-Related Costs per 14-Day CEI Event Among Patients With CEIs in the Overall mTNBC Cohort (**B**) Abbreviations: CEI, clinical event of interest; ILD, interstitial lung disease; IRR, infusion-related reaction; LOT, line of therapy; mTNBC, metastatic triple negative breast cancer; PPPM, per patient per month.

Mean all-cause healthcare costs PPPM were slightly higher among patients with ($17 944) vs without ($16 304) CEIs within 12 months of any LOT initiation. The difference ($1640) was driven mainly by higher mean inpatient costs among patients with vs without CEIs ($1180 higher). Mean all-cause costs PPPM were $1802 and $5333 higher in patients with vs without CEIs during LOT 1 and LOT 2, respectively (**Figure 3A**). Within outpatient medical categories, the largest differences in mean costs PPPM were ancillary/other outpatient services during LOT 1 ($489) and infused or injectable drugs during LOT 2 ($1921). Detailed results are presented in **Supplemental Table S4**. The mean (SD) cost per 14-day CEI event for any CEI among all patients (n = 2717) was $7950 ($9983) and among patients with CEIs (n = 2067) was $10 450 ($10 241). Among the CEIs, sinus bradycardia (n = 18 patients) had the highest mean (SD) cost per 14-day CEI event at $22 354 ($18 944) per patient, followed by ILD (n = 10 patients) at $14 663 ($19 482). Mean costs per 14-day CEI for alopecia, IRRs, stomatitis/mucositis, hematological, gastrointestinal, and hepatic events, and fatigue were within a narrow range from $11 000 to $13 500 (**Figure 3B**).

## DISCUSSION

This retrospective healthcare claims study used recent data from approximately 3000 patients with mTNBC in the US to assess treatment patterns and estimate costs of care and incidence of clinical events. Our study focused on patients with mTNBC with the greatest unmet need for more therapeutic options, namely those who did not receive immunotherapy.^11^ Clinical events, including potential adverse events indicative of treatment tolerability, were observed in 3 out of 4 patients during treatment.

To our knowledge, there is no published literature describing treatment patterns or economic outcomes in a similarly defined population of patients with mTNBC who did not receive PD-L1 immunotherapy, but we can corroborate our findings with treatment guidelines and overall data on patients with mTNBC. Chemotherapy was received by nearly all patients (99%) in our study, which is consistent with US treatment guidelines for mTNBC.^23^ In a study reporting mTNBC treatment patterns using electronic health record data from 2011 to 2021, the authors found that the most common first-line treatments were capecitabine monotherapy (19.0%), carboplatin and gemcitabine combination therapy (9.1%), and cyclophosphamide and doxorubicin combination therapy (9.1%); the most common second-line treatments were capecitabine monotherapy (13.9%), eribulin monotherapy (10.1%), and paclitaxel monotherapy (9.2%).^24^ Capecitabine, gemcitabine, platinum-based chemotherapy, cyclophosphamide, and doxorubicin were also observed as the top 5 treatment regimens in LOT 1 and LOT 2 in our study. Consistent with the electronic health record study, we found no dominant treatment regimen in LOT 1 or LOT 2. Our study described treatment patterns through the end of 2023; therefore, treatment regimens included a small number of patients who received more recently approved drugs, such as sacituzumab govitecan. However, treatment patterns were mostly driven by chemotherapy used as monotherapy or in combination with other chemotherapies.

Our study findings also confirm the high economic burden of mTNBC reported in previously published studies. The mean all-cause healthcare cost PPPM for our mTNBC cohort was $14 245 during follow-up, with higher costs during LOT 1 ($17 809) and LOT 2 ($19,797). Given that the patients were predominantly treated with chemotherapy and not with PD-L1 inhibitors, our findings may be compared with a claims database study using data from 2011 to 2017 (before the 2019 approval of atezolizumab) which reported mean all-cause total healthcare costs of $17 727 PPPM for patients with mTNBC during first-line chemotherapy, including a mean total BC-related treatment cost of $10 322 PPPM, compared with BC-related costs in our study of $11 333.^25^

Our study also investigated the incidence of clinical events and their associated costs of management. Adverse events are expected during treatment for advanced BC and effective management is needed to maintain patient quality of life.^26^ To that end, most patients (76%) had evidence of clinical events during treatment. For example, 50% and 45% of patients had evidence of hematological and gastrointestinal events, respectively, during any LOT (these CEIs thereby making the biggest total contribution to overall CEI-related costs). This highlights the real-world unmet need for more tolerable alternatives to chemotherapy for patients with mTNBC.

CEI-related costs accounted for 35% of all-cause costs during follow-up and a larger proportion of all-cause costs in LOT 1 (51%) than LOT 2 (41%). Outpatient medical costs accounted for more than 50% of CEI-related costs. Across all treatment categories, mean all-cause healthcare costs PPPM were numerically higher among patients with vs without CEIs within 12 months of LOT initiation, which suggests that patients with CEIs experience both a greater clinical and economic burden than those without CEIs. While sinus bradycardia and ILD had the highest mean 14-day CEI-related costs, these CEIs were the least common and further data are needed in larger cohorts to confirm these findings.

Our study has several strengths including the use of a comprehensive database for assessing the study outcomes and a study period with contemporaneous data (up to year 2023). PharMetrics Plus® captures adjudicated medical and pharmacy claims data with patient-level enrollment information, providing a comprehensive longitudinal view of treatment patterns and healthcare costs for all interactions with the healthcare system covered by the patient’s health plan benefit. Our study has limitations inherent to a retrospective study using claims data, including potential misclassification of diagnosis, data entry errors, and lack of clinical data to define BC subtypes, identify treatment indications, and improve capture of CEIs, particularly lower grade CEIs, which may not be coded in the claims database. PharMetrics Plus® is a database of commercially insured individuals; therefore, our results may not be generalizable to uninsured and underinsured populations or patients with Medicare or Medicaid. Our cohort included only patients who received at least 1 LOT during the follow-up period therefore, our outcomes are applicable to a treated patient population. We did not have data on BC stage at initial diagnosis to define de novo vs recurrent mTNBC or data to define genetic/molecular subtypes, which are characteristics of mTNBC that might have HCRU and cost-of-care implications.

Similar to other claims-based studies,^18,19,27^ this study used prescription fill and drug administration dates to estimate the LOT durations; however, they may be underestimated if some treatments were not captured in the claims database or overestimated due to the allowable gap period between treatment claims. Since CEI-related costs were a subset of BC-related costs in this study, CEI-related costs may have included BC-related medication costs on the same claim. This could have inflated CEI-related costs, especially for the relatively small number of patients who received more costly targeted therapies that are associated with specific CEIs. In a sensitivity analysis (results not shown), we found that BC treatment costs accounted for less than 20% of CEI-related costs among patients during chemotherapy.

Our study relied on treatment-based proxies to identify a cohort of patients with TNBC. While this is a common approach for claims-based studies,^18,19,28–30^ there is a risk of misclassification, and our cohort may have included patients with HR+ and/or HER2+ BC subtypes. Likewise, non-receipt of PD-L1 immunotherapy is an imperfect proxy for ineligibility to receive immunotherapy, particularly during the interval between the start of our cohort (March 2017) and FDA approval of the first PD-L1 immunotherapy for mTNBC (atezolizumab, in March 2019). Exclusion of patients who received PD-L1 immunotherapy at any time during the study period may have led to some patients being excluded if they had received pembrolizumab in the early stage setting prior to metastatic recurrence. Accordingly, although the proportion of all patients with TNBC who were excluded by treatment proxy (26%) is within the range of the reported prevalence of PD-L1 positivity among patients with mTNBC (20-40%), we would expect that some of the patients in our analysis were eligible for immunotherapy.^11^

## CONCLUSION

In this cohort of patients with mTNBC, most of whom received chemotherapy as standard of care, CEIs presented both a clinical and economic burden, highlighting the need for newer treatments that balance total costs of care with adverse events and clinical benefit. Given the expansion in targeted TNBC treatment options in recent years, up-to-date data are needed to understand real-world treatment patterns, CEIs, and economic burden among patients with mTNBC and where gaps remain in patient management, especially among patients who are ineligible for immunotherapy.

### Disclosures

A.M.N., D.T., J.T., N.Z. employed by IQVIA, which received consulting fees from AstraZeneca to conduct this study. M.B. has received consulting fees from AstraZeneca, Daiichi Sankyo, Gilead, Eli Lilly, and Pfizer. S.C. is an employee of AstraZeneca Pharmaceuticals Ltd (UK) and holds stock options in AstraZeneca. T.T. has received consulting fees from AstraZeneca, Genentech, Pfizer, Merck, Daiichi Sankyo, Gilead Sciences, GlaxoSmithKline, Tersera, Stemline Therapeutics, Exact Sciences, BioNTech SE, Veracyte, Aktis Oncology, Ellipses Pharma.

## Supplementary Material

Online Supplementary Material

## Data Availability

The data used in this study were obtained from IQVIA’s proprietary database under a data use agreement. These data are not publicly available due to licensing restrictions and confidentiality agreements.
