# Supplementary material for: Treatment Patterns, Clinical Events, and Costs of Care for Patients With Triple Negative Metastatic Breast Cancer: A Retrospective US Claims Database Study
Source: J Health Econ Outcomes Res. 2025 Nov 6;12(2):183–92. doi: 10.36469/001c.144499 (PMC12596037; doi:10.36469/001c.144499)
Supplement: Online Supplementary Material [file jheor_2025_12_2_144499_309124.pdf]

Journal of Health Economics and Outcomes Research

Online Supplementary Material

Treatment Patterns, Clinical Events, and Costs of Care for Patients with Triple-Negative Metastatic Breast Cancer: A Retrospective US Claims Database Study. *JHEOR*. 2025;12(2):183-192. doi:10.36469/jheor.2025.144499

Table S1: Line of Therapy Definitions

Figure S1: Patient Selection Criteria and Attrition

Table S2: Time (Days) from Start of Any LOT to the First Evidence of CEI During Treated Follow-up and by LOT

Figure S2: Median Time from Start of Any LOT to the First Evidence of CEI (Top 5) for mTNBC Cohort, by LOT

Table S3: All-Cause, BC-Related, and CEI-Related Costs PPPM in the mTNBC Cohort During the Minimum 3-Month Follow-up Period

Table S4. All-Cause Costs PPPM in the mTNBC Cohort by LOT and Evidence of CEIs Within 12 Months of LOT Initiation

This supplementary material has been provided by the authors to give readers additional information about their work.

This is an open-access article distributed under the terms of the Creative Commons Attribution 4.0 International License (CCBY-4.0). View this license's legal deed at http://creativecommons.org/licenses/by/4.0 and legal code at http://creativecommons.org/licenses/by/4.0/legalcode for more information.

Table S1. Line of Therapy Definitions

LOT	Rule
Initiation of LOT	1. First occurrence of any anticancer drug(s) on or after index date (earliest evidence of mBC based on diagnosis codes)<br>2. If 1 administration occurred and then no additional administration occurred within 60 days of the same LOT, the drug was ignored from the LOT.
≥LOT 1	1. A universal LOT regimen was made up of the first identified drug, and all other drugs received within the following 28 days. Therefore, there was a 29-day window for identifying whether the LOT is a monotherapy or combination therapy.<br>2. Any mBC treatment of interest occurring between LOT 1 initiation date and 28 days from LOT initiation date is considered part of a combination therapy.
LOT Advance	1. LOT advanced when a new drug was given that was not part of the current 29-day LOT regimen<br>2. LOT advanced when there was a treatment gap of >360 days for the same drug if the regimen contains an oral drug (ie, allow for drug holiday of up to 360 days)<br>3. LOT advanced when there was a treatment gap of >180 days for the same drug if the regimen contains a parenteral (non-oral) drug (ie, allow for a drug holiday of up to 180 days). Parenteral drugs are usually given by injection or infusion.
Does not advance the LOT	1. Drug drops from within a regimen did not advance the LOT.<br>2. Drugs added within 28 days of the LOT start date did not advance the LOT.<br>3. Drug dosing changes did not advance the LOT. This study did not evaluate drug dosing.<br>4. The start of paclitaxel, docetaxel, or nab-paclitaxel 100 days after a regimen that contains cyclophosphamide + doxorubicin starts did not advance the LOT.<br>5. Switches between paclitaxel, docetaxel, and nab-paclitaxel that occurred within 30 days of the start of whichever came first. Therefore, if a switch occurred among these drugs while the other drugs in the regimen continue then it did not advance the LOT.<br>6. Trastuzumab biosimilars could be used interchangeably with trastuzumab and did not advance the LOT.<br>7. Change from cisplatin to carboplatin did not advance the LOT.
Others	1. If a regimen started on or after metastatic date (index date), then regimen was a metastatic LOT.<br>2. Tamoxifen and toremifene drugs could be used interchangeably and did not advance the LOT when they were substituted with one another.<br>3. Letrozole, anastrozole, and exemestane drugs could be used interchangeably and did not advance the LOT when they were substituted with one another.<br>4. Leuprolide, megestrol, and goserelin were treated as supportive agents and did not advance the LOT when added.<br>5. Doxorubicin liposomal could be used interchangeably with doxorubicin and was treated the same as doxorubicin.<br>6. The 360-day gap for oral drugs and 180-day gap for non-oral drugs overrides the gap rule for tamoxifen, toremifene, letrozole, anastrozole, estradiol and exemestane, such that the regimen end date and the start of a non-tamoxifen, toremifene, letrozole, anastrozole, exemestane drug (ignoring the supportive agents megestrol, goserelin, leuprolide) advanced the LOT so long as it was after the 180-day gap for non-oral drugs or 360-day gap for oral drugs for the same drug.

Abbreviations: LOT, line of therapy; mBC, metastatic breast cancer.

Figure S1. Patient Selection Criteria and Attrition

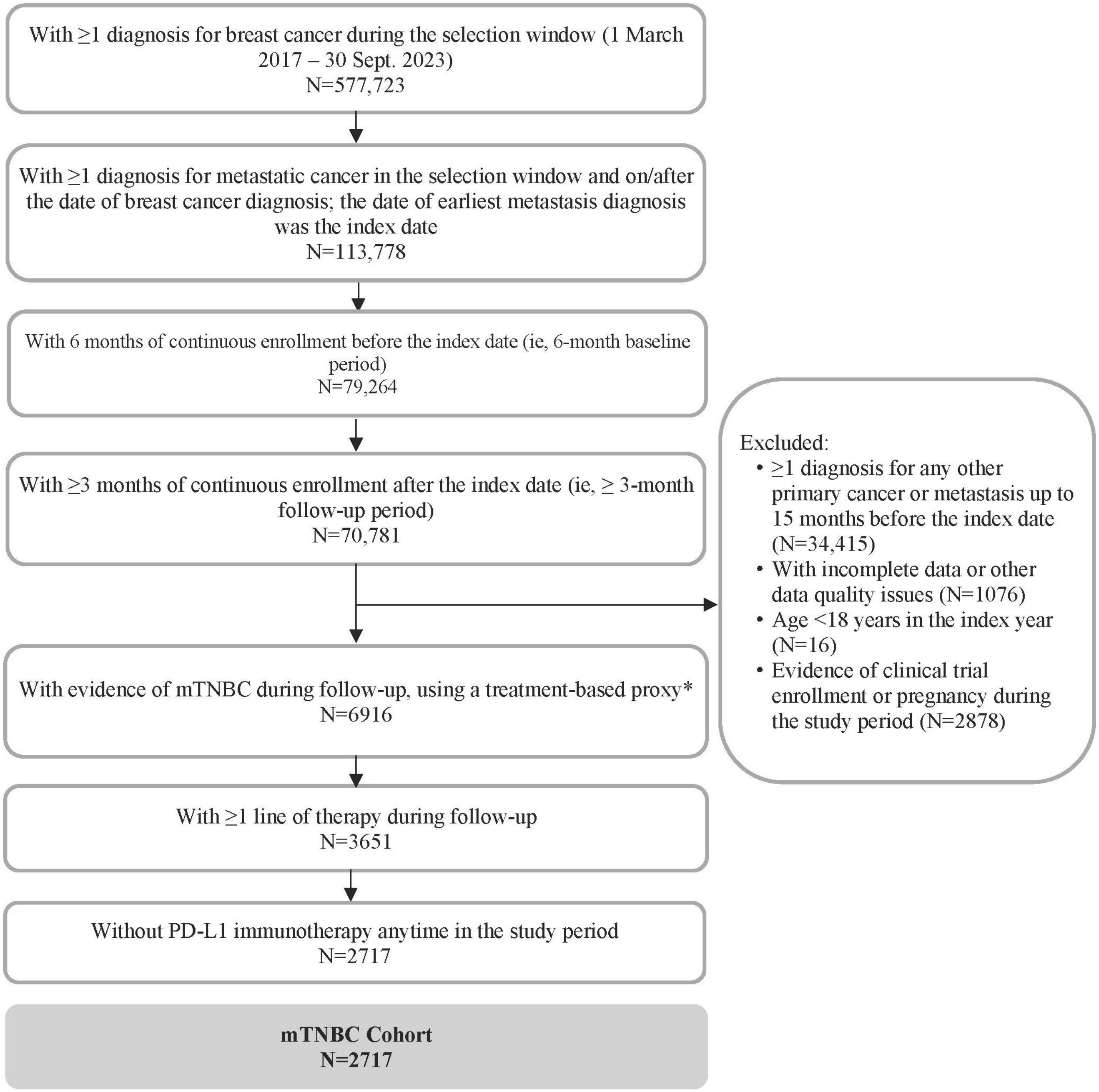

Abbreviations: mTNBC, metastatic triple negative breast cancer; PD-L1, programmed death-ligand 1.
*A treatment-based proxy was used to establish TNBC status, whereby patients with claims for treatments indicated for HR+/HER2- breast cancer (CDK4/6 inhibitor, mTOR inhibitor, PIK3CA inhibitor, AKT inhibitor), HR+ breast cancer (endocrine therapy) or HER2+ breast cancer (trastuzumab, pertuzumab, margetuximab, ado-trastuzumab emtansine, lapatinib, neratinib, tucatinib) during the study period were excluded.

Table S2. Time (Days) From Start of Any LOT to the First Evidence of CEI During Treated Follow-up and by LOT

Time (Days) from Start of LOT to CEI, Among Patients With ≥1 Event	Overall mTNBC Cohort (N = 2717)	During LOT 1 (N = 2717)	During LOT 2 (N = 731)
Alopecia[a]			
No. with ≥1 event	68	57	7
Mean (SD)	39.9 (40.3)	36.8 (35)	38.7 (49.3)
Median (Q1, Q3)	26.5 (14, 52.5)	24 (14, 50)	36 (5, 42)
Fatigue			
No. with ≥1 event	732	579	169
Mean (SD)	52.2 (91.1)	52.7 (96)	57 (60.6)
Median (Q1, Q3)	32 (14, 64.5)	35 (14, 63)	40 (13, 83)
Gastrointestinal events			
No. with ≥1 event	1,158	1,018	191
Mean (SD)	31.7 (75.8)	30.7 (71.4)	41.9 (59.9)
Median (Q1, Q3)	14 (0, 41)	14 (0, 41)	19 (5, 51)
Stomatitis/mucositis			
No. with ≥1 event	158	136	20
Mean (SD)	42 (45.9)	39.5 (40.9)	33.5 (29.8)
Median (Q1, Q3)	28 (14, 54)	28 (14, 51)	21 (13.5, 48)
Hematological events			
No. with ≥1 event	1260	1097	227
Mean (SD)	33.7 (70.1)	33.9 (72.1)	37.5 (51.4)
Median (Q1, Q3)	14 (1, 45.5)	14 (1, 47)	20 (7, 48)
Hepatic events			
No. with ≥1 event	59	42	11
Mean (SD)	79.2 (173.8)	90.9 (199.5)	43.3 (55.3)
Median (Q1, Q3)	43 (10, 84)	53.5 (21, 84)	30 (0, 49)
Hyperglycemia			
No. with ≥1 event	109	82	26
Mean (SD)	88.1 (163.6)	80.1 (149.9)	85 (86.6)
Median (Q1, Q3)	56 (19, 92)	53 (24, 84)	67 (7, 140)
ILD			
No. with ≥1 event	11	7	2
Mean (SD)	85.1 (61.1)	79 (41.7)	35 (24.0)
Median (Q1, Q3)	83 (27, 123)	85 (27, 123)	35 (18, 52)
IRRs			
No. with ≥1 event	806	632	181
Mean (SD)	56.2 (85.8)	58.3 (86.8)	52.3 (63.1)
Median (Q1, Q3)	33.5 (13, 72)	41 (15, 74)	28 (10, 78)
Ocular events			
No. with ≥1 event	170	127	32
Mean (SD)	97.4 (150.9)	92.3 (141.7)	73.8 (97.5)
Median (Q1, Q3)	61 (25, 105)	63 (30, 105)	36.5 (10, 90)
Rash			
No. with ≥1 event	233	174	62
Mean (SD)	69.2 (100.4)	74 (112.1)	62.9 (53.3)
Median (Q1, Q3)	51 (21, 98)	55.5 (26, 104)	49.5 (18, 99)

Table S2. Time (Days) From Start of Any LOT to the First Evidence of CEI During Treated Follow-up and by LOT

Time (Days) from Start of LOT to CEI, Among Patients With ≥1 Event	Overall mTNBC Cohort (N = 2717)	During LOT 1 (N = 2717)	During LOT 2 (N = 731)
Sinus bradycardia			
No. with ≥1 event	18	13	4
Mean (SD)	69.6 (104.9)	39.5 (71.1)	96.5 (103.6)
Median (Q1, Q3)	20 (8, 62)	14 (8, 35)	75 (15.5, 177.5)

Abbreviations: ILD, interstitial lung disease; IRR, infusion-related reaction; Q1, first quartile; Q3, third quartile.

Figure S2. Median Time from Start of Any LOT to the First Evidence of CEI (Top 5) for mTNBC Cohort, by LOT

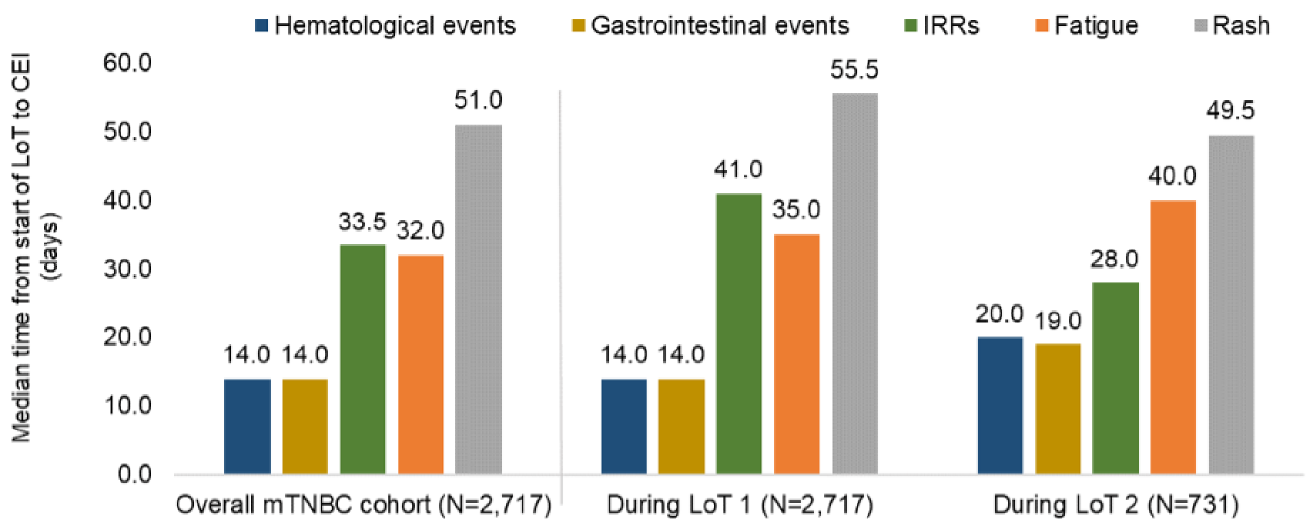

Abbreviations: CEI, clinical event of interest; IRR, infusion-related reaction LOT, line of therapy; mTNBC, metastatic triple-negative breast cancer.

Table S3. All-Cause, BC-Related, and CEI-Related Costs PPPM in the mTNBC Cohort During the Minimum 3-Month Follow-up Period

**Healthcare Costs PPPM (2024 USD)**	**mTNBC Cohort (N = 2717)**		
	**All-Cause**	**BC-Related**	**CEI-Related**
Total costs			
Mean (SD)	$14 245 ($12 776)	$11 333 ($11 267)	$4949 ($7748)
Median (Q1, Q3)	$11018 ($5657, $19 088)	$8513 ($3710, $15 632)	$507 ($782, $6388)
Subgroups of total costs			
Inpatient			
Mean (SD)	$2518 ($6900)	$2080 ($6238)	$1,806 ($5916)
Median (Q1, Q3)	$0 ($0, $2360)	$0 ($0, $497)	$0 ($0, $931)
Outpatient pharmacy			
Mean (SD)	$1268 ($3457)	$489 ($2131)	$452 ($1753)
Median (Q1, Q3)	$190 ($33, $887)	$0 ($0, $153)	$11 ($3, $76)
Outpatient medical			
Mean (SD)	$10459 ($9406)	$8764 ($8833)	$2691 ($4475)
Median (Q1, Q3)	$8045 ($3901, $14 100)	$6433 ($2707, $12 212)	$1102 ($288, $3347)
Subgroups of outpatient medical costs			
Physician office visits			
Mean (SD)	$477 ($477)	$326 ($294)	$51 ($98)
Median (Q1, Q3)	$369 ($232, $584)	$253 ($129, $434)	$14 ($0, $60)
ED visits			
Mean (SD)	$129 ($348)	$54 ($234)	$29 ($159)
Median (Q1, Q3)	$0 ($0, $107)	$0 ($0, $0)	$0 ($0, $0)
Laboratory and pathology services			
Mean (SD)	$577 ($668)	$455 ($597)	$94 ($201)
Median (Q1, Q3)	$344 ($140, $781)	$228 ($77, $603)	$26 ($8, $94)
Radiology			
Mean (SD)	$2068 ($2869)	$1704 ($2477)	$158 ($566)
Median (Q1, Q3)	$1097 ($445, $2441)	$871 ($285, $2017)	$24 ($0, $116)
Infused or injectable drugs			
Mean (SD)	$3241 ($5348)	$2968 ($5034)	$2025 ($3976)
Median (Q1, Q3)	$1382 ($227, $4150)	$1185 ($131, $3773)	$613 ($5, $2359)
Outpatient surgical services			
Mean (SD)	$1498 ($2166)	$1197 ($1958)	$53 ($304)
Median (Q1, Q3)	$803 ($2894, $1848)	$528 ($130, $1451)	$0 ($0, $0)
Ancillary/other outpatient services			
Mean (SD)	$2469 ($3536)	$2060 ($3389)	$281 ($734)
Median (Q1, Q3)	$1545 ($747, $3004)	$1154 ($465, $2542)	$19 ($0, $219)

Abbreviations: BC, breast cancer; CEI, clinical event of interest; ED, emergency department; mTNBC, metastatic triple-negative breast cancer; PPPM, per patient per month; Q1, first quartile; Q3, third quartile; USD, US dollars.

Table S4. All-Cause Costs PPPM ($) in the mTNBC Cohort by LOT and Evidence of CEIs Within 12 Months of LOT Initiation

**Healthcare Costs PPPM (2024 USD)**	**Overall mTNBC Cohort (N = 2717)**		**During LOT 1 (N = 717)**		**During LOT 2 (N = 731)**	
	**With CEIs (N = 2273)**	**Without CEIs (N = 444)**	**With CEIs (N = 2251)**	**Without CEIs (N = 466)**	**With CEIs (N = 599)**	**Without CEIs (N = 132)**
Total costs						
Mean (SD)	17 944 (16 263)	16 304 (16 417)	18 118 (19 254)	16 316 (16 409)	20 760 (26 486)	15 427 (15 484)
Median (Q1, Q3)	13 740 (8476, 21 796)	11 599 (6737, 20 794)	13 516 (8289, 21 796)	11 801 (6744, 20 769)	12 899 (5610, 26 601)	11 123 (3463, 23 048)
Subgroups of total costs						
Inpatient						
Mean (SD)	1604 (6385)	424 (2896)	1472 (6623)	566 (4046)	3162 (17 344)	709 (3927)
Median (Q1, Q3)	0 (0, 0)	0 (0, 0)	0 (0, 0)	0 (0, 0)	0 (0, 0)	0 (0, 0)
Outpatient pharmacy						
Mean (SD)	2075 (5244)	2329 (6401)	2086 (5923)	2313 (6549)	3032 (7409)	3467 (7041)
Median (Q1, Q3)	302 (31, 1748)	116 (14, 1586)	119 (22, 1336)	90 (14, 1537)	707 (89, 2514)	1013 (153, 3110)
Outpatient medical						
Mean (SD)	14 264 (14 484)	13 551 (15 813)	14 560 (17 780)	13 437 (15 556)	14 566 (19 843)	11 251 (14 571)
Median (Q1, Q3)	10 806 (5796, 17 654)	9353 (3998, 17 298)	10 911 (5720, 17 654)	9359 (4090, 16 879)	7646 (2825, 18 873)	5930 (1347, 15 891)
Subgroups of outpatient medical costs						
Physician office visits						
Mean (SD)	585 (594)	485 (545)	590 (608)	485 (533)	628 (761)	403 (396)
Median (Q1, Q3)	457 (301, 692)	345 (218, 576)	458 (299, 704)	349 (222, 592)	448 (276, 748)	276 (153, 527)
ED visits						
Mean (SD)	179 (631)	47 (266)	172 (642)	47 (263)	199 (667)	68 (307)
Median (Q1, Q3)	0 (0, 85)	0 (0, 0)	0 (0, 0)	0 (0, 0)	0 (0, 0)	0 (0, 0)
Laboratory and pathology services						
Mean (SD)	489 (718)	345 (606)	523 (956)	356 (618)	556 (1033)	373 (618)
Median (Q1, Q3)	232 (82, 605)	120 (51, 422)	226 (78, 636)	123 (53, 425)	179 (74, 573)	101 (37, 463)
Radiology						
Mean (SD)	1464 (5608)	1340 (8205)	1274 (5624)	1239 (7985)	2004 (5326)	1362 (3229)
Median (Q1, Q3)	191 (0, 964)	0 (0, 297)	102 (0, 684)	0 (0, 306)	362 (0, 1904)	53 (0, 1018)
Infused or injectable drugs						
Mean (SD)	8233 (11 853)	8490 (12 196)	8483 (14 706)	8486 (12 063)	8172 (17 365)	6251 (13 229)
Median (Q1, Q3)	5051 (603, 10 887)	4596 (41, 11 629)	5034 (309, 11 069)	4702 (34, 11 582)	267 (0, 10 480)	10 (0, 5633)
Outpatient surgical services						
Mean (SD)	451 (1163)	244 (847)	442 (1378)	236 (771)	607 (1687)	541 (1768)
Median (Q1, Q3)	21 (0, 362)	0 (0, 60)	0 (0, 257)	0 (0, 55)	17 (0, 446)	0 (0, 177)
Ancillary/other outpatient services						
Mean (SD)	2863 (2984)	2601 (3220)	3077 (3325)	2588 (3160)	2401 (3187)	2253 (3548)
Median (Q1, Q3)	2013 (1118, 3707)	1781 (917, 3145)	2142 (1199, 3976)	1776 (948, 3181)	1443 (480, 3174)	940 (108, 2989)

Abbreviations: BC, breast cancer; CEI, clinical event of interest; ED, emergency department; LOT, line of therapy; mTNBC, metastatic triple-negative breast cancer; PPPM, per patient per month; Q1, first quartile; Q3, third quartile; USD, US dollars.
